# Probing the Allosteric Modulation of P-Glycoprotein: A Medicinal Chemistry Approach Toward the Identification of Noncompetitive P-Gp Inhibitors

**DOI:** 10.1021/acsomega.2c08273

**Published:** 2023-03-14

**Authors:** Cátia A. Bonito, Ricardo J. Ferreira, Maria-José. U. Ferreira, Fernando Durães, Emília Sousa, Jean-Pierre Gillet, M. Natália D. S. Cordeiro, Daniel J. V. A. dos Santos

**Affiliations:** † LAQV@REQUIMTE, Department of Chemistry and Biochemistry, Faculty of Sciences, University of Porto, Rua do Campo Alegre, Porto 4169-007, Portugal; ‡ Red Glead Discovery AB, Medicon Village, Scheelevägen 8, Lund 223 63, Sweden; § Research Institute for Medicines (iMed.ULisboa), Faculty of Pharmacy, Universidade de Lisboa, Av. Prof. Gama Pinto, Lisbon 1649-003, Portugal; ∥ Interdisciplinary Centre of Marine and Environmental Research (CIIMAR) & Laboratory of Organic and Pharmaceutical Chemistry, Department of Chemical Sciences, Faculty of Pharmacy, University of Porto, Rua Jorge Viterbo Ferreira 228, Porto 4050-313, Portugal; ⊥ Laboratory of Molecular Cancer Biology, URPhyM, NARILIS, Faculty of Medicine, University of Namur, Namur 5000, Belgium; # CBIOS-Center for Research in Biosciences & Health Technologies, Lusófona University, Campo Grande, 376, Lisboa 1749-024, Portugal

## Abstract

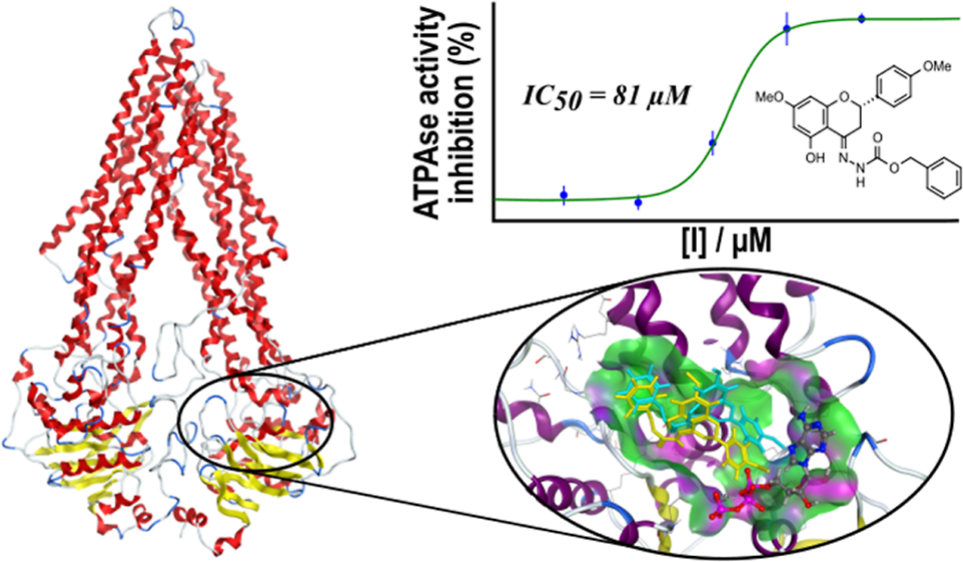

A medicinal chemistry approach combining *in silico* and *in vitro* methodologies was performed aiming at identifying and characterizing putative allosteric drug-binding sites (aDBSs) at the interface of the transmembrane- and nucleotide-binding domains (TMD-NBD) of P-glycoprotein. Two aDBSs were identified, one in TMD1/NBD1 and another one in TMD2/NBD2, by means of *in silico* fragment-based molecular dynamics and characterized in terms of size, polarity, and lining residues. From a small library of thioxanthone and flavanone derivatives, experimentally described to bind at the TMD-NBD interfaces, several compounds were identified to be able to decrease the verapamil-stimulated ATPase activity. An IC_50_ of 81 ± 6.6 μM is reported for a flavanone derivative in the ATPase assays, providing evidence for an allosteric efflux modulation in P-glycoprotein. Molecular docking and molecular dynamics gave additional insights on the binding mode on how flavanone derivatives may act as allosteric inhibitors.

Over-expression of membrane efflux pumps as P-glycoprotein (P-gp, ABCB1) is tightly related to the multidrug resistance (MDR) phenomenon in cancer cells,^1−3^ and chemotherapy failure.^4^ Therefore, modulating drug efflux by P-gp is, currently, still one of the most promising strategies to beat MDR in cancer. P-gp architecture consists of two transmembrane domains (TMDs) and two cytoplasmic nucleotide-binding domains (NBDs) organized in a pseudo-2-fold symmetry. Four short intracellular coupling helices (ICHs), located between the transmembrane helices (TMHs) 2/3 (ICH1-NBD1), 4/5 (ICH2-NBD2), 8/9 (ICH3-NBD2), and 10/11 (ICH4-NBD1), mediate the communication between both domains through noncovalent interactions.^5,6^ The drug-binding pocket (DBP) lies within the TMDs,^7^ and it is capable of interacting with several structurally unrelated scaffolds. To date, three distinct generations of P-gp modulators were categorized, but all of them failed to demonstrate their efficacy and safety in the clinical environment.^8,9^ Furthermore, the development of more selective and effective P-gp modulators, using structure-based approaches, was also hampered by the polyspecificity of the DBP.^7,10^ Thus, novel strategies for P-gp efflux modulation are extremely important to reverse MDR in cancer cells.

Recently, specific motifs within the ABC architecture—the ICHs—have been described as a possible target for small molecules. Targeting such motifs has several advantages because they (i) are highly conserved among the ABCB transporter family and (ii) are involved in the propagation of the conformational changes from the inward- to outward-facing conformations, thus acting as TMD-NBD signal-transmission interfaces. Specifically for the members of the subfamily B involved in drug efflux (where P-gp is included, along with ABCB5), the ICHs also seem to play important roles in the activity, folding, and maturation of the transporter. Targeting such domains by small molecules have the advantage of avoiding competition with the DBP, thus vastly reducing the adverse effects identified previously during the clinical trials of past MDR modulator generations. If proven successful for the case of P-gp, this novel approach can also be applicable to other members of the ABC transporter superfamily.^11^

Possible allosteric drug-binding site (s) (aDBSs) have been proposed at the ICH–NBD interfaces, able to bind small molecules that potentially block the TMD–NBD signal-transmission, responsible for driving conformational changes leading to efflux.^11^ Small molecules such as flavonoids,^12,13^ thioxanthones,^14^ and 1,4-dihydropyridine derivatives^15−17^ were predicted to interact in these regions. However, the first study that clearly identified a possible drug-binding region next to the ICHs was performed by Kim *et al.* in the *Arabidopsis thaliana* P-gp homologue.^18^ The authors predicted that the ABCB1-dependent auxin transport inhibitors 2-[4-(diethylamino)-2-hydroxybenzoyl] benzoic acid and 1-*N*-naphthylphthalamic acid (BUM and NPA, respectively) bind in a pocket located between the ICHs and the Q-loops of NBDs. Furthermore, while NPA interacts in both NBDs, BUM preferentially binds at the NBD2 and in an additional region between NBD1 and NBD2.

Therefore, aiming at accurately identifying any putative aDBSs at the ICH-NBD interfaces (Figure 1), a computational fragment-based drug discovery (cFBDD) approach was undertaken. The fragments were derived (i) from molecules that are experimentally described to bind at the ICH–NBD interfaces such as BUM/NPA^18^ or flavonoids, for example narigenin^13^ and (ii) from *in silico* studies of molecules that also have one energetic minimum at similar regions, for example, tariquidar, nicardipine, isoxazol-DHP, and morphine^11^ (although experimentally a photolabeling site for dihydropyridines is also proposed to exist in close proximity to such motifs).^15−17^ That said, preference was given to aromatic systems in which additional moieties (*e.g.*, carboxylic acid, alcohols, amides, and nitriles) and substitution patterns were chosen to maximize the coverage of the intended chemical space. The fragments obtained from each molecule are depicted in Table S1 of the Supporting Information file.

Several simulated-annealing molecular dynamics (MD) simulations were performed only using the cytoplasmic portion of the N- and C-terminals (concerning NBD1 and NBD2, respectively). For each NBD, five MD systems were built, each one comprising six fragment types and five copies of each fragment (for a total of 30 fragments), by randomly inserting them in the surrounding water environment using the GROMACS *gmx insert-molecules* tool. The fragments included in each MD system are depicted in Table S2 of the Supporting Information file. Five replicates of each system were performed in a total of 25 MD runs per NBD. Then, occupancy volumetric maps were generated from the MD trajectories to identify possible hotspots at the ICH–NBD interfaces using the *VolMap* tool in VMD (see Materials and Methods section in the Supporting Information file).

Overall, the occupancy maps (Figure 2) showed two important hotspots at the ICH–NBD interfaces in each NBD comprising (i) both ICHs and the A-loop motif of the respective NBD or (ii) the ICH4/2 (NBD1 or NBD2, respectively) and the Q-loop/Walker A (WA) motifs in each NBD. In addition, while most fragments preferred to bind between the ICHs and the A-loop of NBD1, a more equal distribution of the fragments was observed for NBD2.

**Figure 1:**
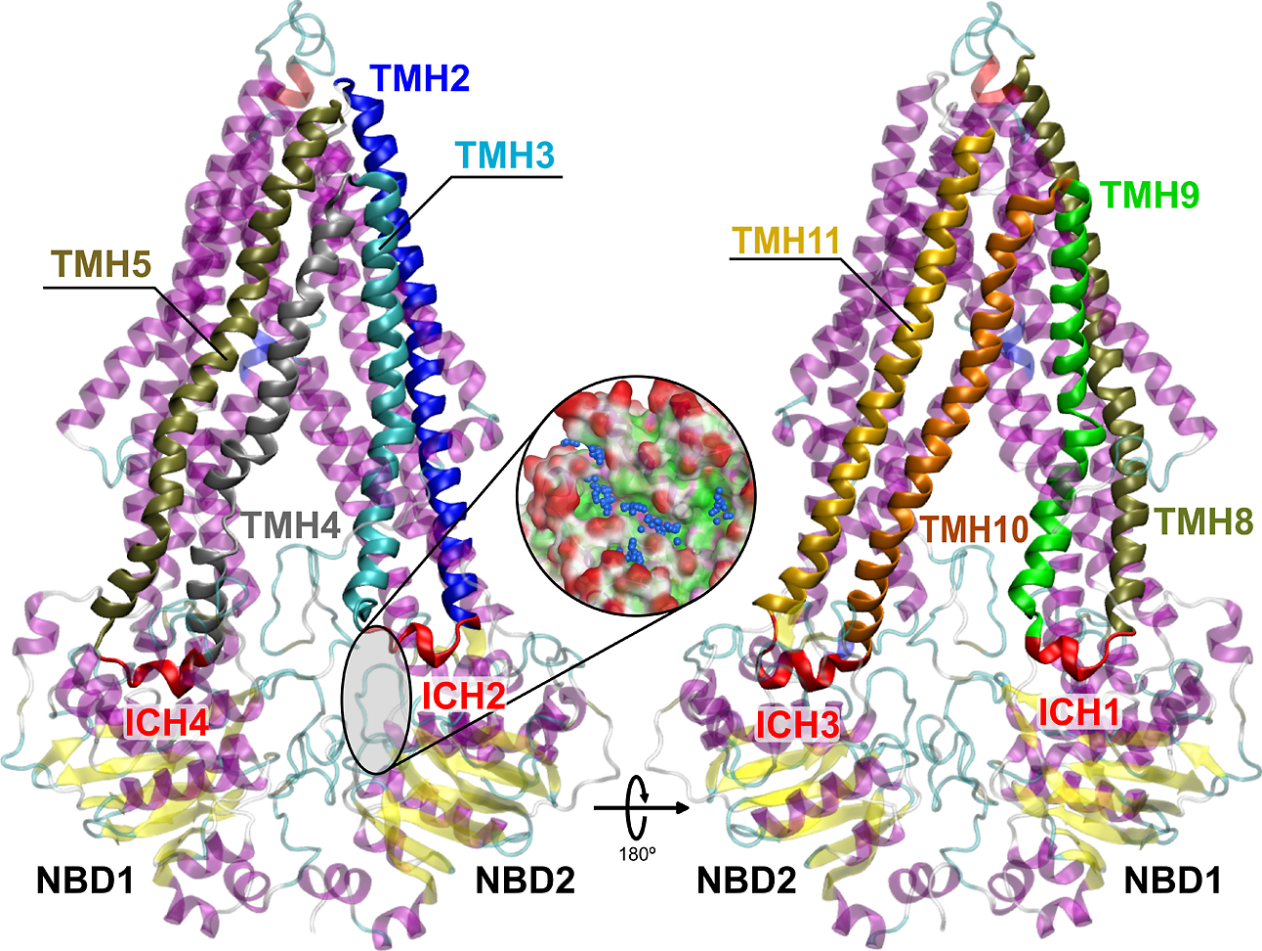
Structural representation of the human P-gp in an inward-facing conformation (transparent rendering helices in purple, beta sheets in yellow, turns in cyan, and coils in white). TMHs directly connected to each ICH (red) are labeled and colored differently for clarification. The putative allosteric site at NBD2 is identified in the central motif (blue dummy spheres were obtained using the *Site Finder* module available in MOE 2019.01).

**Figure 2:**
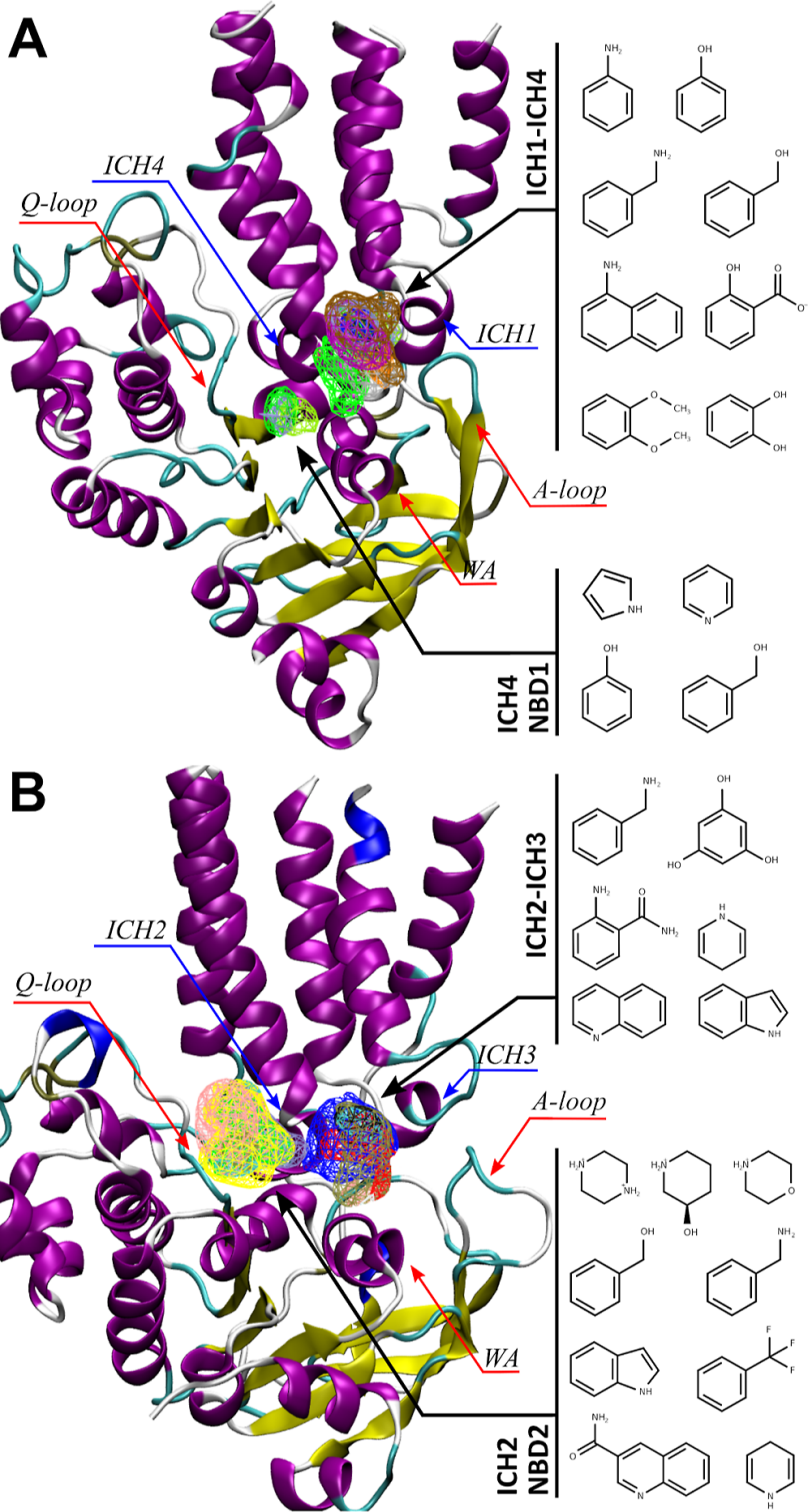
Occupancy volumetric maps and identified fragments at each hotspot for (A) NBD1 and (B) NBD2. The color code for the volumetric occupancy maps can be found in the Supporting Information, Table S2.

Apart from occupancy maps, obtained by MD simulations, the corresponding fragment types within each hotspot were also considered crucial to identify which chemotypes are preferred. Concerning the hotspot located in-between ICHs and closer to the A-loop motif of NBD1, six-membered aromatic ring systems are preferred, but fused-ring systems are also tolerated, with a preference toward aromatic amines and hydroxyl and methoxy groups (often in an *ortho* substitution pattern for the latter). Oppositely, heterocyclic rings such as pyrrole and pyridine, together with the benzylamine and benzylalcohol substructures, preferentially bound at the second hotspot, located closer to the ICH4/Q-loop/WA motifs.

On the other hand, a higher heterogeneity was observed for NBD2. Concerning the hotspot next to both ICHs and closer to the A-loop motif, single- (1,4-dihydropyridine) or fused-heterocyclic ring systems (quinoline, indole) are tolerated, as well as amine/amide groups and hydroxyl substituents (the latter in a *meta* substitution pattern). Interestingly, at the ICH2/Q-loop/WA hotspot, a preference for positively charged fragments was identified, namely, the piperazine, 3-hydroxy-piperidine, or morpholine scaffolds (not observed in NBD1), together with other moieties such as benzylamine, benzyl alcohol, 1,4-dihydropyridine or trifluoromethylphenyl moieties, and fused-ring systems (3-amidoquinolines, indoles).

These results indicate that although the same fragments’ database was applied to both NBDs, different scaffolds were observed in the equivalent hotspots of the NBDs, thus suggesting an apparent degree of specificity between NBD1 and NBD2. More importantly, as some fragments were derived from known binders such as BUM (phenol, 2-hydroxybenzoate) and NPA (naphtalen-1-amine),^18^ isoxazole-DHP (1,4-dihydropyridine)^15−17^ or naringenin (benzene-1,2-diol, benzene-1,3,5-triol),^12,13^ were also found to bind to these hotspots, and the results are found to be in good agreement with the experimentally available information.

Following the hotspot identification and to better characterize these novel regions, lining residues, pocket volume, residue distribution, and mean polarity were assessed using the EPOS^BP^ and MOE packages and compared with the modulator site (M-site), located at the top of the DBP (see Materials and Methods section in the Supporting Information file).^7^ The results suggest that the two hotspots are probably part of a larger aDBS, herein named as aDBS1 for NBD1 and aDBS2 for NBD2. The mean polarity and residue distribution showed that both aDBSs are more polar than the M-site, containing 47 ± 1% of polar residues, against 30% of the M-site. Both sites also seem to have similar volumes (683 ± 21 Å^3^), but when concerning the solvent accessible surface area, aDBS2 seems to be slightly more exposed (∼231 Å^2^*vs* ∼198 Å^2^ for aDBS1).

Afterward, an evaluation on drug binding to this specific location was performed by molecular docking using the previously published human P-gp homology model.^5^ This is a fast technique that can be swiftly used to assess the ability of small molecules in binding to the proposed aDBS. Based on the scaffolds found in each hotspot at the ICH–NBD interfaces, a small in-house library of thioxanthone^14^ and flavanone derivatives^19,20^ was selected for this purpose (Supporting Information, Table S3). Additionally, since BUM and NPA were experimentally predicted to bind in a pocket located between the ICHs and Q-loop motif of NBDs,^18^ these compounds were also included as references in our docking studies. The docking box was defined to include all regions between ICHs and the whole ATP-binding site (see Materials and Methods section in the Supporting Information file). Ten docking poses were generated per molecule, but to simplify the results only the top-ranked binding energies (Δ*G*_dock_) at each aDBS will be described.

Overall, all compounds tested showed favorable Δ*G*_dock_ values in both NBDs (Supporting Information, Tables S4 and S5), similar to those observed at the M-site, ranging from −5.8 kcal/mol (BUM, NBD1) to −8.5 kcal/mol (compound **15**, NBD2), thus indicating that both aDBSs found are hypothetically druggable, that is, capable of accommodating molecules with favorable binding energies. Moreover, the analysis of the protein–ligand contacts confirmed that the tested molecules overlapped the occupancy maps in both NBDs, interacting with most of the lining residues (Tables S3 and S4) identified and corroborating the location of the aDBS in each NBD.

However, with regard to the NBD1, most of the compounds tested, including BUM and NPA, were found to protrude from the hotspot next to the ICH4/Q-loop/WA motifs and thus partially overlapping the phosphate groups of ATP (or the coordinating magnesium ion). On the contrary, most of the molecules tested in NBD2, including BUM and NPA, did not overlap the ATP-binding site. Yet, such results are in good agreement with previous studies concerning flavonoids, wherein a partial overlapping between a flavonoid-binding region and the ATP-binding site was inferred from experimental data.^12,13^ Additionally, when compared with thioxanthones, flavanone derivatives also have higher probabilities of occupying the hotspot in-between ICH2 and ICH3, which can be prone to “lock” ICH2 and ICH3 together. Altogether, all of the above data are consistent with the existence of an allosteric drug-binding site in each TMD–NBD interface, vicinal to that where ATP binds, involving the ICHs and the A-loop, Q-loop, and WA motifs of the respective NBD. In addition, the favorable binding energies obtained for all molecules in the molecular docking studies led us to assume that, indeed, both aDBSs are expected to be druggable.

As most of the compounds tested in our docking studies bind at this vicinal region, with or without partially overlapping the ATP-binding site, ATPase assays were conducted to evaluate their effect on drug-stimulated P-gp ATPase activity. Herein, the tested compounds were incubated with 200 μM verapamil, a P-gp substrate that stimulates the P-gp ATPase activity.^21,22^ The ATPase assays were carried out in recombinant human P-gp membranes using the Pgp-Glo Assay System (Promega, The Netherlands) and according to the manufacturer’s experimental protocol^23^ (see Materials and Methods section in the Supporting Information file). First, an initial screening of the in-house libraries of thioxanthone and flavanone derivatives was performed. Two reference compounds, BUM—experimentally validated as an allosteric inhibitor of the *A. thaliana* P-gp homologue^18^—and the triterpene spiropedroxodiol—a potent and competitive efflux inhibitor^24^—were also included in the ATPase assays for comparison purposes. The basal P-gp ATPase activity and verapamil-stimulated ATPase activity in the absence or presence of compounds were estimated against the sodium orthovanadate (Na_3_VO_4_)-treated samples, which is a strong P-gp ATPase inhibitor,^2^ in accordance with the Pgp-Glo Assay System technical bulletin. The data were obtained in relative light units (RLUs), but for clarity purposes, the results were normalized using as reference the basal P-gp ATPase activity values (set to 1.0) (Figure 3) (Supporting Information, Table S6 and Figures S1, S2). Data showed that all tested compounds (except the thioxanthone **TX3** and the flavanones **7** and **14**) were able to inhibit the verapamil-stimulated ATPase activity. Herein, while most thioxanthones had a weak activity on inhibiting ATPase, compounds **5**, **16**, **17**, **20**, **23**, **24**, **27**, and **28** from the flavanone derivatives set were classified as moderate-to-good ATPase inhibitors. The reference compounds BUM and spiropedroxodiol were also found to inhibit the verapamil-stimulated ATPase activity but with distinct potencies. As anticipated, BUM (expected to have an allosteric effect on P-gp efflux) had a stronger impact in decreasing the verapamil-stimulated ATPase activity than spiropedroxodiol, a competitive efflux P-gp inhibitor expected to bind at the TMD–NBD allosteric site with lower affinities (−6.7 kcal/mol), when compared with those reported for both M- and R-sites (−9.7 kcal/mol).^24^ It is also conceivable that spiropedroxodiol has a weaker effect than BUM due to the absence of an intact membrane surrounding P-gp required for lipophilic compounds such as spiropedroxodiol to gain access to the drug-binding sites^11,22^ located in-between the transmembrane domains.

**Figure 3:**
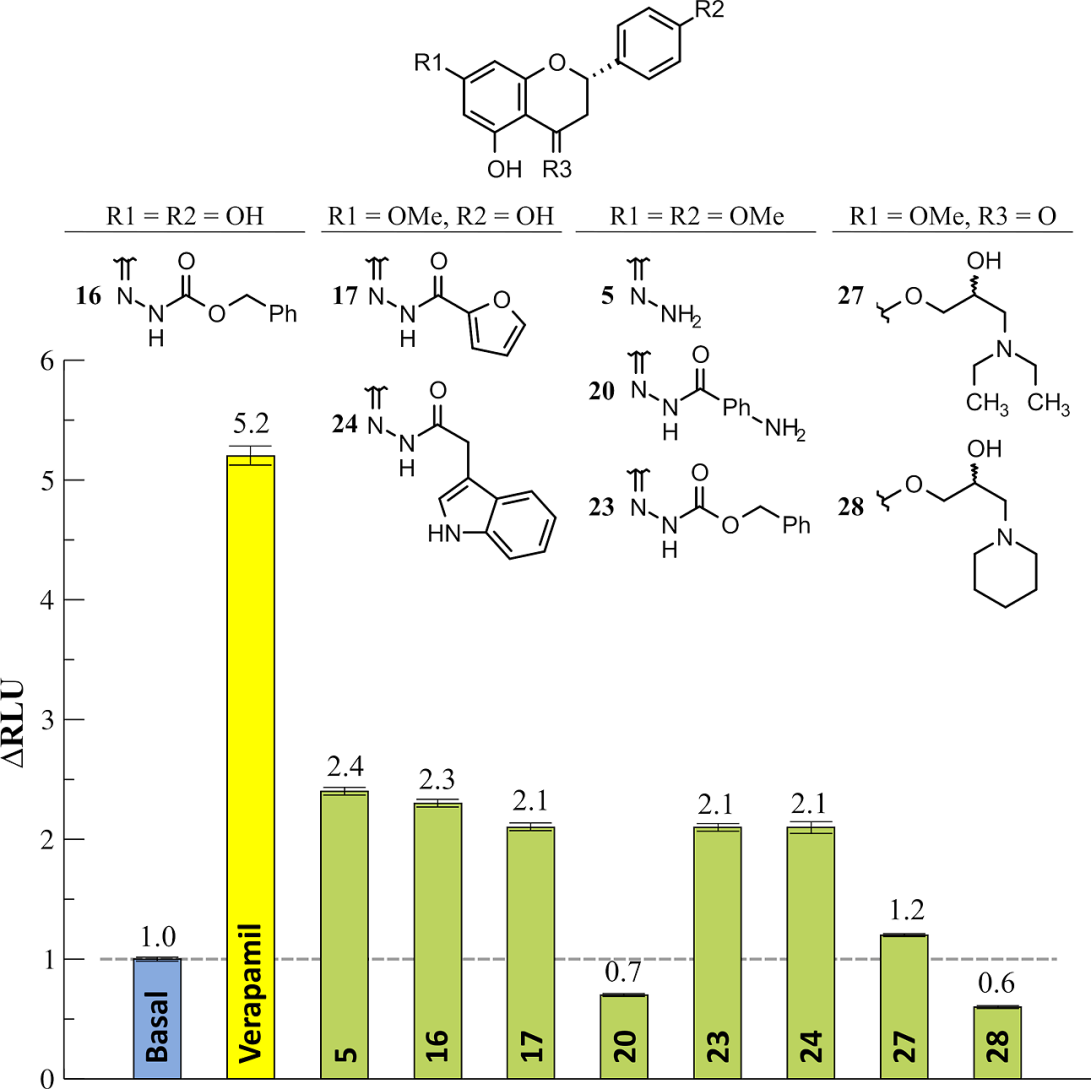
Normalized ATPase activity values (only for the compounds chosen for IC_50_ determination) (more details on the ΔRLU can be found in the Supporting Information file, Section 5). The chemical structures of selected compounds are depicted and drawn in MarvinSketch.

Interestingly, all other compounds with lower ATPase inhibition rates (compounds **1–4**, **6–15**, **18**, **19**, **21**, **22**, **25**, and **26**) were previously described as competitive efflux modulators in the rhodamine-123 (R123) accumulation assay,^19,20^ with fluorescence activity ratios (FARs) ranging from 2.10 (**7**) up to 10.67 (**8**) at 2.0 μM. The only exceptions are compounds **27** (FAR, 4.06; Δ*RLU*, 1.2) and **28** (FAR, 3.08; Δ*RLU*, 0.6). This reinforces our hypothesis that the selected flavanones are expected to act through a different mechanism, most probably as allosteric inhibitors.

Next, the compounds that had the highest inhibitory effect in verapamil-stimulated ATPase activity (Figure 3) were chosen for calculating the respective half maximal inhibitory concentration (IC_50_) values (Table 1). Test compounds were evaluated at a range of concentrations for their capability to inhibit 200 μM of verapamil-stimulated ATPase activity according to the experimental protocol. The obtained IC_50_ results allowed the identification of the flavanone derivative **23** (Figure 4) as a promising hit for novel P-gp allosteric inhibitors with an IC_50_ of 81 ± 6.6 μM. Despite registering some degree of inhibition, compounds **16** and **17** failed to produce a dose–response curve. Concerning compounds **20** and **28**, these are classified as inhibitors but with an IC_50_ above the maximum concentration used in the assay (200 μM).

**Figure 4:**
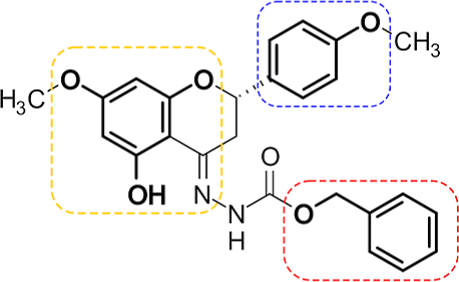
Structure of compound **23**. Matching fragments with cFBDD are highlighted (red, phenylmethanol; blue, *p*-methoxyphenyl; yellow, benzene-1,3,5-triol).

**Table 1:** IC_50_ Values Determined From ATPase Inhibition Experiments (*n* = 3)

compound	class	IC_50_ (μM)
**5**	inhibitor	189 ± 34
**16**	non-inhibitor	
**17**	non-inhibitor	
**20**	inhibitor	>200
**23**	inhibitor	81 ± 6.6
**24**	inhibitor	>200
**27**	inhibitor	>200
**28**	inhibitor	>200

As flavonoids are described to interact preferentially with NBD2,^13^ multiple MD runs were performed using the top-ranked docking pose of compound **23** obtained at NBD2 as a starting configuration (see Materials and Methods section in the Supporting Information file).

Five replicates were carried out, in a total of 300 ns of simulation time, and their relative free-energies of binding (Δ*G*_MD_) were estimated using the *g_mmpbsa* tool.^25^ The results showed two possible binding modes (BMs) for compound **23** with similar binding energies (Figure 5). The first one (BM_1_) was obtained in three out of five simulations, having a Δ*G*_MD_ of −26 ± 2.4 kcal/mol, with A259/A260 (ICH2) and Q-loop residue Q1118 as residues with higher contributions for binding (Supporting Information, Table S7).

**Figure 5:**
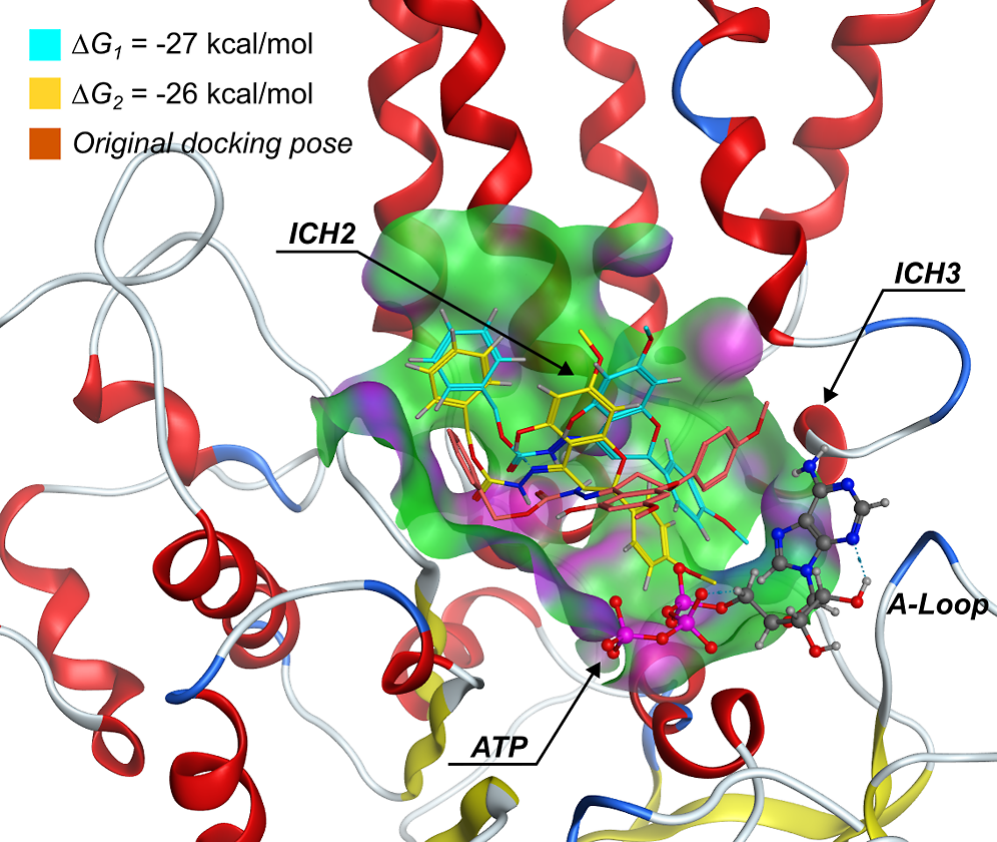
Averaged final configurations for compound **23** obtained from MD simulations (yellow, *n* = 3; cyan, *n* = 2). The original docking pose is depicted in brown. Pocket surface is colored by *ActiveLP* (pink, H-bonding; green, hydrophobic; blue, mild polar). ATP pose was derived from PDB ID: 6C0V and is depicted for comparison purposes only.

The second binding mode (BM_2_) was found to occur in the remaining two simulations, in which compound **23** was found between ICH2 and ICH3, with an average Δ*G*_MD_ of −27 ± 0.3 kcal/mol. Herein, residues L258, A259, I261 (ICH2), F804, and T811 (ICH3) had the highest contribution for ligand binding. Quite interestingly, F804 corresponds to the phenylalanine residue at position 792 (F792) in the *A. thaliana* sequence and was the residue specifically described to be experimentally involved in BUM binding (Supporting Information, Table S7).

Altogether, the above data allowed us to infer that compound **23** might not have a preferential binding mode, and thus we hypothesize that the observed ATPase inhibitory activity results, instead, from two different mechanisms. Herein, the analysis of BM_1_ for compound **23** suggests that the signal transmission mechanism may be impaired by disrupting the contacts between the ICH2 and the Q-loop of NBD2. Accordingly, some studies identified the glutamine located within the Q-loop (Q1118) as having an important role for ATP-binding/hydrolysis and Mg^2+^ binding as mutations in this residue blocked drug-stimulated ATPase activity.^26^ Furthermore, the Q1118 residue is also described as a key residue in coupling events occurring in the DBP to ATP binding.^27,28^

More interestingly, BM_2_ additionally suggests an agreement with a mechanism proposed by Loo and Clarke.^29^ As demonstrated by the authors, the cross-linking of the mutant A259C/W803C (residues located at ICHs 2 and 3, respectively) inhibited the P-gp drug-stimulated ATPase activity possibly by impairing the conformational changes that occurred at the ICH2/ICH3 interface upon ATP binding.^29^ Therefore, we hypothesize that compound **23** may induce a similar “locking” of ICHs 2 and 3, impairing the signal transmission mechanism triggered by verapamil at the DBP by inhibiting the conformational changes promoted by ATP binding.

Finally, the possibility of compound **23** to act as a noncompetitive inhibitor is reinforced by a previous published work made in our group using the same flavanone derivative dataset. Quite interestingly, compound **23**—corresponding to compound **31** in previous work^19^—had much higher cytotoxicity in ABCB1-overexpressing L5178Y (L5178Y-MDR) than in parental cell lines (7.25 μM *vs* > 100 μM, respectively), but it was unable to promote R123 accumulation in resistant cells (FAR of 0.94 and 1.13 at 2.0 and 20 μM, respectively). Yet, it was still able to synergistically enhance doxorubicin cytotoxicity. These findings led the authors to suggest that this particular compound modulates P-gp drug efflux by a different mechanism.^19^ Herein, and based on our findings, we hypothesize that compound **23** is able to act as an allosteric inhibitor by targeting the ICHs–NBD2 interface and impairing signal transmission, thus blocking the efflux-related conformational changes that ultimately led to drug efflux.

Summarizing, in this letter, we have provided further evidence that (i) an allosteric binding site at the TMD–NBD interface exists within the P-gp architecture, as demonstrated by the binding of small fragments derived from known molecules, (ii) the proposed binding site is in agreement with previous literature, slightly overlapping the ATP-binding site but with different characteristics in each of the NBDs, (iii) the tested thioxanthone and flavanone derivatives bind to the proposed binding site with favorable affinities, thus inferring that the aDBSs are apparently druggable, and (iv) the flavanone scaffold may be a suitable building block for the design of novel allosteric P-gp modulators.

The above results demonstrate the “proof-of-concept” of allosteric efflux modulation of ABC transporters, at least when concerning P-gp. This opens future perspectives toward the development of a new generation of P-gp efflux modulators, also enabling the usage of computational techniques such as fragment expansion and high-throughput virtual screening to expedite and guide the discovery of new P-gp modulators.

## Data Availability

Data including (i) volumetric occupancy maps for all fragments found at both NBD1 and NBD2 allosteric sites, (ii) docking results for all thioxantones and flavanone derivatives, and (iii) topology/trajectory files for the MD simulations of compound 23 are available for download at (http://chemistrybits.com).

## Supporting Information Available

Materials and methods, molecular fragments used in MD simulations, chemical structures of experimentally evaluated compounds, contacts between top-ranked docking pose and NBDs, ΔRLU and normalized RLU values for thioxanthones and flavanone derivatives, and detailed MD results (PDF)
