# Supplementary material for: Probing the Allosteric Modulation of P-Glycoprotein: A Medicinal Chemistry Approach Toward the Identification of Noncompetitive P-Gp Inhibitors
Source: ACS Omega. 2023 Mar 14;8(12):11281–7. doi: 10.1021/acsomega.2c08273 (PMC10061618; doi:10.1021/acsomega.2c08273)
Supplement: Supplementary file 1 — ao2c08273_si_001.pdf [file ao2c08273_si_001.pdf]

SUPPORTING INFORMATION

"Probing the allosteric modulation of P-glycoprotein: A medicinal chemistry approach towards the identification of non-competitive P-gp inhibitors."

Cátia A. Bonito,[†] *Ricardo J. Ferreira,*[‡] *Maria-José. U. Ferreira,*[£] *Fernando Durães,*[§] *Emília Sousa,*[§] *Jean-Pierre Gillet,*[¥] *M. Natália D. S. Cordeiro,*[†] *Daniel J. V. A. dos Santos*[‖*]

[†]LAQV@REQUIMTE, Department of Chemistry and Biochemistry, Faculty of Sciences, University of Porto, Rua do Campo Alegre, 4169-007 Porto, Portugal; [‡]Red Glead Discovery AB, Medicon Village, Scheelevägen 8, 223 63 Lund, Sweden; [£]Research Institute for Medicines (iMed.ULisboa), Faculty of Pharmacy, Universidade de Lisboa, Av. Prof. Gama Pinto, 1649-003 Lisbon, Portugal; [§]Laboratory of Organic and Pharmaceutical Chemistry, Department of Chemical Sciences, Faculty of Pharmacy, University of Porto, Rua Jorge Viterbo Ferreira 228, 4050-313 Porto, Portugal; [¥]Laboratory of Molecular Cancer Biology, URPhyM, NARILIS, Faculty of Medicine, University of Namur, 5000 Namur, Belgium; [†‖]CBIOS- Center for Research in Biosciences & Health Technologies, Lusófona University, Campo Grande, 376, 1749-024 Lisboa, Portugal.

TABLE OF CONTENTS

Material and Methods.

Table S1: Fragments obtained from each molecule.

Table S2: Fragments included in each MD system.

Table S3: Experimentally evaluated compounds.

Table S4: NBD1 residue interactions with top-ranked docked poses of all tested molecules and total number of contacts.

Table S5: NBD2 residue interactions with top-ranked docked poses of all tested molecules and total number of contacts.

Table S6: ΔRLU values for first-screening compounds.

Figure S1: Normalized RLU values for first-screening of compounds (all compounds, flavonoids).

Figure S2: Normalized RLU values for first-screening of compounds (all compounds, thioxanthones).

Table S7. Protein-residue contacts, residue contribution to binding and hydrogen-bonding.

MATERIAL AND METHODS

1. Computational Fragment-Based Drug discovery.

1.1 Fragments selection and parametrization. Fragments were obtained from predicted ICH–NBD binders,[1-3] drawn in MarvinSketch v.19.18 and exported to MOE[4] software for further protonation and minimization using the MMFF94x[5] force-field (adjusting hydrogen and lone pairs by default). Following, each fragment was parametrized in the PRODRG[6] online server and manually curated, according to the GROMOS96 54a7 force field,[7] adjusting partial charges to the AM1-BCC scheme[8] calculated with Antechamber 1.27.[9]

1.2 Construction of the Molecular Dynamics (MD) systems. The previously published human P-gp homology model, obtained from the murine P-gp crystallographic structure (PDB ID: 4Q9H), refined and equilibrated using MD simulations, was used in all simulations (the whole system can be downloaded at http://chemistrybits.com/?smd_process_download=1&download_id=720). For ~~(for~~ additional details on the homology modeling, systems preparation and model validation we refer to the original publication from Bonito and co-workers.[10] Only the spatial coordinates for the cytoplasmic portion of the N- and C-terminal (concerning NBD1 and NBD2, respectively) were used in this study (with an RMSD of 3.8 Å when superimposed with the cryo-EM human P-gp structure, PDB ID: 6QEX). The preparation of the initial structures was performed using VMD and MOE software packages. Each NBD was inserted in a simulation box with dimensions *xyz* of 10 × 10 × 9 nm^3 and PBC conditions were applied to all dimensions. Following, all systems were solvated with an adequate number of SPC water molecules. For each NBD, five MD systems were built (*Table S2*), each one comprising six fragment types and five copies of each (for a total of 30 fragments), by randomly inserted them in the surrounding water environment using *gmx insert-molecules* module available in GROMACS[11] software. Any overlapping waters were automatically removed, and any charge excess were neutralized by adding an adequate number of counter-ions (sodium or chlorine).

1.3. Simulated-annealing molecular dynamics simulations (saMD). After an energy minimization step, a short 10 ps *NVT* ensemble run followed by a 5-ns *NpT* ensemble MD simulations were performed for equilibrating temperature (303 K) and pressure (1 bar), respectively, while keeping all protein atoms spatially restrained. Following, a 50 ns MD run was performed for each system, using a simulated annealing protocol applied to the fragments, solvent and ions to decrease the probability of non-specific binding to each of the NBDs. Herein, two heating cycles (303 K to 323 K) were performed, increasing the temperature during 1 ns (t = 0 ns and t = 25 ns) and decreasing over a period of 3 ns (t = 2 ns and t = 27 ns). Due to the simulated annealing procedure, the protein´s alpha carbons

were kept restrained throughout the whole MD simulation to prevent unfoldings (1000 kJ/mol nm^2). For each system, five replicates were performed (25 saMD systems per NBD, in a total of 1.25 μs of simulation time per NBD).

1.4 Analysis and identification of allosteric drug-binding site(s). The last 30 ns of each replicate were concatenated using *gmx trajcat* tool available in GROMACS to obtain a single trajectory file of 100 ns per NBD. An isosurface cut-off of 0.1 was used to fine-tune the generated occupancy maps. Visual inspection of the fragments' occupancy maps was performed in VMD, and only those in close vicinity of each TMD-NBD interface were considered for the identification of possible binding sites next to the ICH domains. Finally, the properties of each putative aDBS such as lining residues, pocket volume, residues distribution and mean polarities were evaluated using the $EPOS^{BP}$ [12] software (default parameters), and compared with the modulator site (M-site).

2. Molecular Docking studies. Molecular docking were performed in the AutoDock VINA v1.1.2[13] in both isolated NBDs. Considering the location of the selected occupancy maps, a docking box was defined to include all regions between the ICHs and the whole ATP-binding site, with grid dimensions *xyz* of 18.75 × 18.75 × 18.75 $Å^3$ (NBD1) or 18.75 × 22.50 × 18.75 $Å^3$ (NBD2), and centered at the *xyz* dimensions of 40.27 × 53.20 × 55.31 $Å^3$ (NBD1) or 63.40 × 47.61 × 55.70 $Å^3$ (NBD2), respectively. The database consisted in small in-house libraries of flavonoids (n = 28)[2,14] and thioxanthones (n = 8).[15] Additionally, due to experimental evidences that BUM and NPA bind in a region located at the ICH-NBD interfaces, these molecules were also included in the docking studies as references. Ten docking poses were generated for each ligand. Molecules were manipulated as previously stated, but saved in the PDB format. For further usage in the AutoDock VINA docking software, both receptor (P-gp) and ligands were converted to the PDBQT format in AutoDockTools v1.5.6rc.[16] Visual inspection of the docking poses was made in MOE, and the protein-ligand interactions were calculated using *binana.py*[17] and LigPlot[18] software packages.

3. Free-energy calculations and analysis of compound 23. After a short energy minimization run to minimize clashes between the ligand and the protein, a 100 ns MD run was performed using the top-ranked docking pose of compound **23** at the NBD2, using the final configuration of the NBD2 after the saMD simulations. From the initial MD simulation, four replicates of 50-ns MD run each were performed by selecting distinct snapshots along the simulation (based on the RMSD evolution during the first MD run), namely at 40-, 70- and 90-ns MD simulation run. Only the last 30-ns of MD simulation were considered as production run and used for analysis. The visual inspection of the

binding mode(s) was performed in MOE, relative free-energies of binding (ΔG_{MD}) were calculated using the *g_mmpbsa* tool,[19] residue contact frequencies were estimated with *g_contacts*[20] and *gmx hbond* to assess hydrogen bonding.

4. MD simulation parameters. All MD simulations were done with GROMACS v2016.6 package. All *NVT* equilibration runs were performed at 303 K using the Velocity-rescale (V-rescale)[21] thermostat. The Nosé-Hoover[22,23] thermostat and the Parrinello-Rahman[24] barostat for temperature (303 K) and pressure (1 bar), respectively, were applied in all *NpT* runs. Pressure equilibration was achieved through a isotropic pressure coupling, with the systems' compressibility set to 4.5 × 10-5 bar^{-1}. All bond lengths were constrained using the LINCS[25,26] or SETTLE[27] (for water molecules) algorithms. The Particle Mesh Ewald (PME) with cubic interpolation[28,29] was employed, with a cut-off radius of 12 Å for both electrostatic and van der Waals interactions and an FFT grid spacing of 0.16 for long range electrostatics. Group-based and Verlet[30] cut-off schemes were applied for the calculation of non-bonded interactions on CPU or GPU, respectively.

5. Experimental studies: ATPase assays

5.1 Compounds

A small in-house library of thioxanthone (n = 8) and flavanone (n = 28) derivatives were used to evaluate the effect of such compounds in P-gp ATPase activity using PgpGlo P-gp ATPase assays (Promega, The Netherlands). The compounds were tested at the initial concentration of 200 µM (thioxanthones) and 100 µM (flavonoids). Additionally, the compounds BUM (1 mM) and spiropedroxodiol (50 µM) were also included in the ATPase assays as positive controls for allosteric[3] and competitive P-gp inhibition[31] mechanisms, respectively. All compounds were dissolved in DMSO.

5.2 ATPase assays — general concepts

The ATPase activity of the P-gp ATP-dependent drug efflux pump was measured by using the PgpGlo Assay Systems kit (Promega, The Netherlands) according to the manufactures' recommendation. Briefly, this assay relies on the ATP dependence of the light-generating reaction of firefly luciferase using recombinant human Pgp in a cell membrane fraction.[32] The recombinant human P-gp in cell membrane fraction (25 µg/well) was incubated with 5mM of MgATP for a period of 40 minutes at 37 °C. The reaction is stopped by adding the luciferase-based ATP Detection Reagent. At this stage, luciferase reaction is initiated, and the luminescence signal is produced. Luminescence changes in direct proportion to the unmetabolized ATP concentration (ATP not consumed by P-gp).

Reactions where no ATP is consumed generate the brightest signals (e.g. sodium orthovanadate; Na_3VO_4), while reactions where ATP has been consumed have relatively lower luminescent signals, reflecting lower unmetabolized ATP concentrations (e.g. verapamil).

5.3 ATPase assays layout and analysis of the results

The assay was performed in a 96-well plate layout, which comprised four different sample types: i) non-treated (NT), ii) treated with Na_3VO_4, iii) treated with verapamil (VER), and iv) treated with test compound (TC) in the presence of the substrate verapamil (TC+VER). All the samples were tested in triplicate according to the technical bulletin.

A first screening was performed to evaluate the capacity of compounds to inhibit the P-gp drug-stimulated ATPase activity in the presence of 200 µM of verapamil, a P-gp substrate that stimulates P-gp ATPase activity[33,34] In the following step, the compounds that showed the highest inhibition of the verapamil-stimulated ATPase activity were selected for calculating the respective maximal inhibitory concentration (IC_{50}) values by decreasing the test compounds' concentration using a two-fold dilution, while maintaining verapamil concentration unchanged (200 µM), in agreement with the experimental protocol provided by the manufacturer.

The luminescence signal was read in average relative light units (RLU), and compared with the Na_3VO_4 – a strong P-gp inhibitor[35] – treated samples. The difference between the average luminescent signals from Na_3VO_4-treated samples (RLU_{NA3VO4}) and NT samples (RLU_{NT}) corresponds to the basal Pgp ATPase activity, and calculated as follows:

$$RLU_{Na_3VO_4} - RLU_{NT} = \Delta RLU_{basal}$$

The difference between the average luminescent signals from Na_3VO_4-treated samples (RLU_{NA3VO4}) and VER-treated samples (RLU_{VER}) reflects the VER-stimulated ATPase, and can be determined as follows:

$$RLU_{Na_3VO_4} - RLU_{VER} = \Delta RLU_{VER}$$

Finally, the difference between the average luminescent signals from Na_3VO_4-treated samples (RLU_{Na3VO4}) and TC+VER-treated samples (RLU_{TC}) reflects the ability of TC to inhibit VER-stimulated ATPase activity, and can be calculated by the same equation:

$$RLU_{Na_3VO_4} - RLU_{TC+VER} = \Delta RLU_{TC+VER}$$

Table S1: Fragments obtained from each molecule.

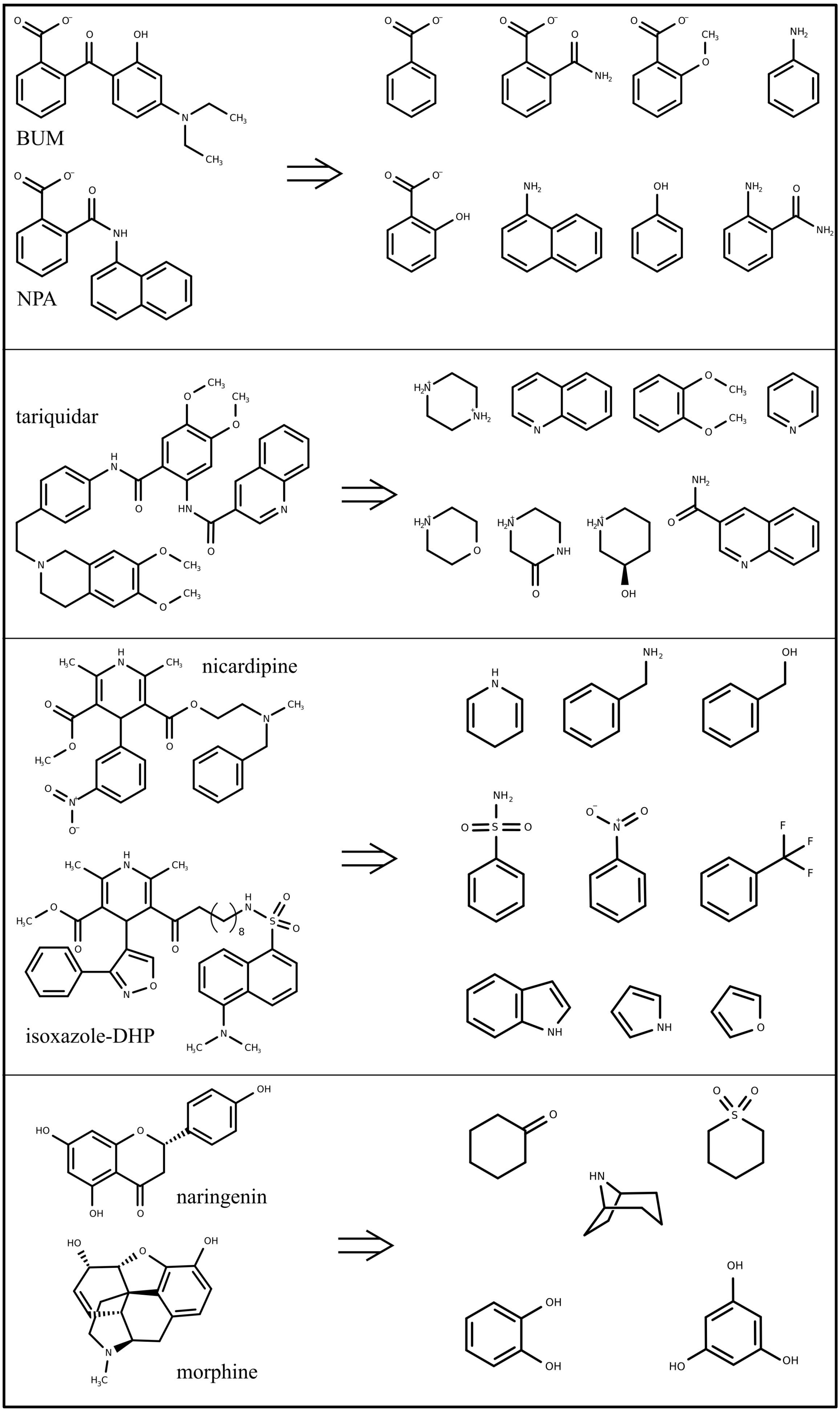

Table S2: Fragments included in each MD system.

System	Fragments (IUPAC name)	Molecular Formula	SMILES	NBD1*	NBD2*
1	Naphthalen-1-amine	$C_{12}H_{15}N$	NC1=CC=CC2=CC=CC=C12		
	Aniline	C_6H_7N	NC1=CC=CC=C1		
	Pyridine	C_5H_5N	C1=CC=NC=C1		
	Benzoate	$C_7H_5O_2^-$	[O-]C(=O)C1=CC=CC=C1		
	Phenol	C_6H_6O	OC1=CC=CC=C1		
	1,4-dihydropyridine	C_5H_7N	C1C=CNC=C1		
2	Benzene-1,2-diol	$C_6H_6O_2$	OC1=C(O)C=CC=C1		
	Benzene-1,3,5-triol	$C_6H_6O_3$	OC1=CC(O)=CC(O)=C1		
	1H-indole	C_8H_7N	N1C=CC2=C1C=CC=C2		
	Nitrobenzene	$C_6H_5NO_2$	[O-][N+](=O)C1=CC=CC=C1		
	1H-pyrrole	C_4H_5N	N1C=CC=C1		
	Quinoline-3-carboxamide	$C_{10}H_8N_2O$	NC(=O)C1=CC2=CC=CC=C2N=C1		
3	2-carbamoylbenzoate	$C_8H_6NO_3^-$	NC(=O)C1=C(C=CC=C1)C([O-])=O		
	1,2-dimethoxybenzene	$C_8H_{10}O_2$	COC1=C(OC)C=CC=C1		
	Furan	C_4H_4O	O1C=CC=C1		
	Phenylmethanamine	C_7H_9N	NCC1=CC=CC=C1		
	Quinoline	C_9H_7N	C1=CC2=CC=CN=C2C=C1		
	Trifluormethylbenzene	$C_7H_5F_3$	FC(F)(F)C1=CC=CC=C1		
4	2-aminobenzamide	$C_7H_8N_2O$	NC(=O)C1=C(N)C=CC=C1		
	2-carboxyphenolate	$C_7H_5O_3^-$	OC1=C(C=CC=C1)C([O-])=O		
	Cyclohexanone	$C_6H_{10}O$	O=C1CCCCC1		
	2-methoxybenzoate	$C_8H_7O_3^-$	COC1=C(C=CC=C1)C([O-])=O		
	Phenylmethanol	C_7H_8O	OCC1=CC=CC=C1		
	Piperazine-1,4-diium	$C_4H_{12}N_2^{2+}$	C1C[NH2+]CC[NH2+]1		
5	Morpholin-4-ium	$C_4H_{10}NO^+$	C1COCC[NH2+]1		
	Benzenesulfonamide	$C_6H_7NO_2S$	NS(=O)(=O)C1=CC=CC=C1		
	piperidin-1-ium-3-ol	$C_5H_{12}NO^+$	OC1CCC[NH2+]C1		
	3-oxopiperazine-1,4-diium	$C_4H_9N_2O^+$	O=C1C[NH2+]CCN1		
	8-azoniabicyclo[3.2.1]octane	$C_7H_{14}N^+$	C1CC2CCCC1[NH2+]2		
	Thiane 1,1-dioxide	$C_5H_{10}O_2S$	O=S1(=O)CCCCC1		

* color code refers to the volumetric occupancy maps in Figure 2.

Table S3: Experimentally evaluated compounds.

TX1	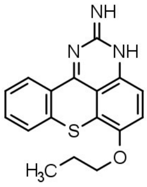	TX2	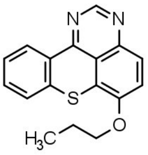
TX3	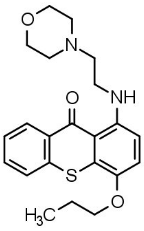	TX4	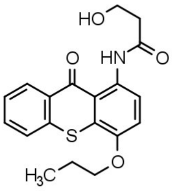
TX5	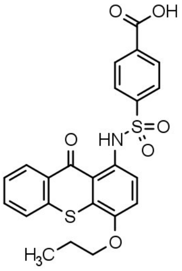	TX6	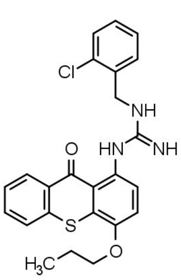
TX7	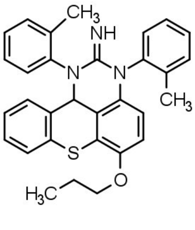	TX8	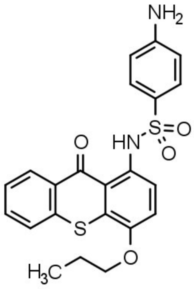
Naringenin	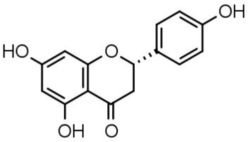	2	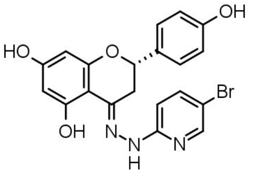
3	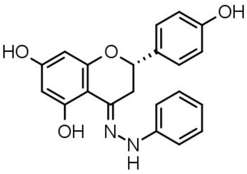	4	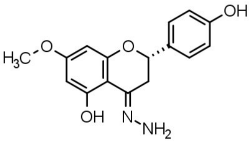

Table S3 (continued)

5	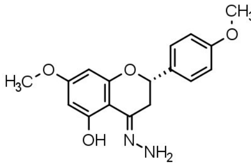	6	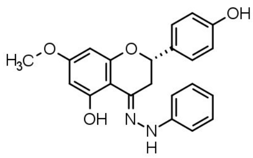
7	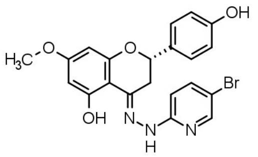	8	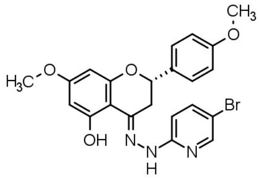
9	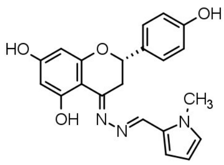	10	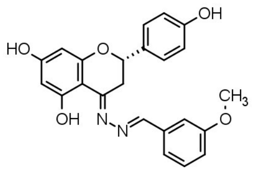
11	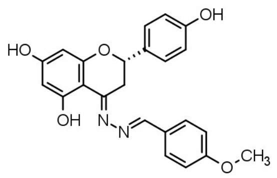	12	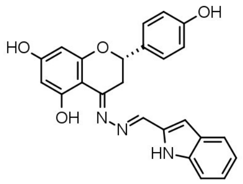
13	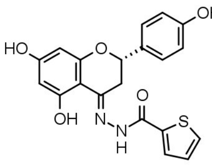	14	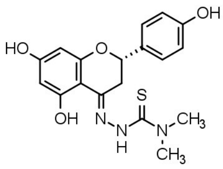
15	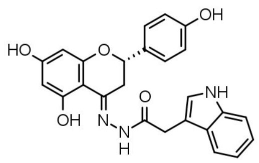	16	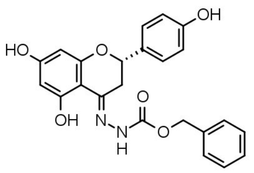
17	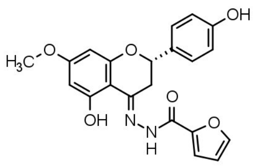	18	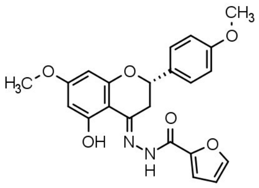
19	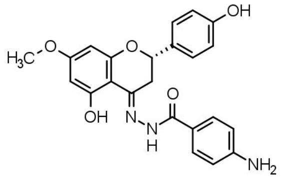	20	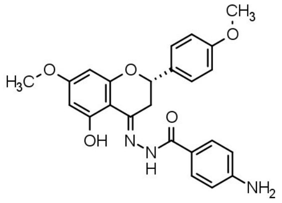

Table S3 (continued)

21	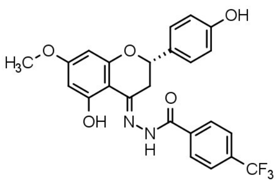	22	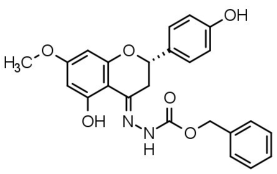
23	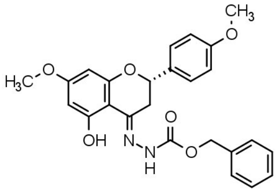	24	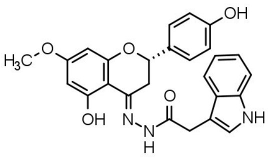
25	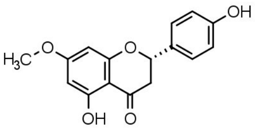	26	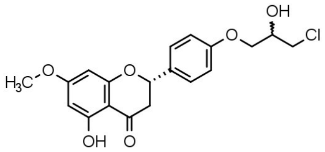
27	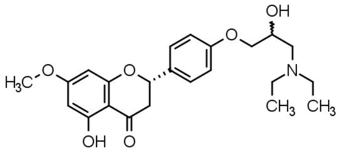	28	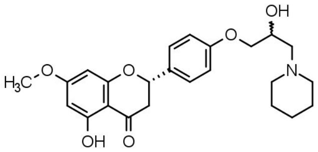
BUM	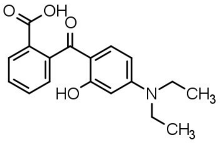	spiropedroxodiol	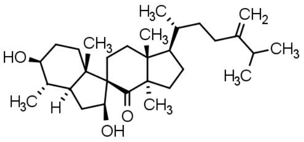

Table S4. NBD1 residue interactions with top-ranked docked poses of all tested molecules and total number of contacts.

Molecule Name		binding energy (kcal/mol)	Hydrogen Bonds		Hydrophobic Contacts		Pi-pi	T-stacking	Cation-pi	Salt Bridges	Contacts	
			ICH	NBD	ICH	NBD					ICH	NBD
REFS	BUM	-5.8	--	S434	E902, N903	V437, D555, I585	--	--	--	--	15	12
	NPA	-7.1	--	--	E902, N903	V437, S474, D555, I585	--	--	--	--	6	17
	Naringenin (**1**)	-6.6	I160	--	I160, F163, V168, E902, F904, R905	--	--	--	R905	--	14	10
	7-OMe-Naringenin (**25**)	-6.5	--	--	E902, N903, T906	V472, V437, S474, D555, I585	--	--	--	--	9	11
Thioxantones	TX1	-7.1	D164	--	I160, F163, D164, V168, I901, E902, F904, R905	--	--	--	R905	--	10	16
	TX2	-6.8	--	--	I160, F163, D164, V168, I901, E902, F904, R905	--	--	--	R905	--	10	16
	TX3	-6.4	R905	S434, E476	--	G430, S434, V437, V472, S474, E476, D555, I585	--	--	--	--	5	22
	TX4	-6.3	--	E476	--	V437, S474, E476, D555	--	--	--	--	4	9
	TX5	-7.1	R905	--	E902, N903, R905, T906	V437, S474, E476, D555, I585	--	--	--	--	15	17
	TX6	-7.8	R905	--	F163, I160, E902, F904, R905	S474, E476	--	--	R905	--	14	11
	TX7	-6.7	--	--	E902, N903	V437, S474, E476, D555	--	--	R905	--	8	10
	TX8	-6.8	--	--	E902, N903, R905, T906	S434, V437, S474, I585	--	--	--	--	17	10
Hydrazones	2	-7.8	--	--	N903	S434, V437, V472, S474, D555, I585	--	--	--	--	7	17
	3	-7.8	--	S434, E476, D555	R905	S434, V437, V472, S474, D555, I585	--	--	R905	--	8	17
	4	-6.7	--	--	N903	V437, V472, S474, D555, I585	--	--	--	--	7	11
	5	-6.4	--	--	E902, N903	V437, V472, S474, L553, L554, D555	--	--	--	--	9	19
	6	-7.6	R905	S434	N903, R905	S434, V437, V472, S474, D555, I585	--	--	R905	--	8	14
	7	-7.5	--	S434, D555	R905	V437, V472, S474, E476, D555, I585	--	--	--	--	7	16
	8	-7.3	--	S434	E902, N903, T906	V437, V472, S474, L553, L554, D555, I585	--	--	--	--	9	17
Azines	9	-7.8	--	E476	R905, T906	Y401, G430, C431, S434, V437, V472, S474, D555, I585	--	--	--	--	8	26
	10	-7.4	N903	S434	N903	G430, S434, V437, S474, D555, I585	--	--	--	--	5	26
	11	-7.5	--	--	E902, N903, R905	R404, V437, S474, E476	--	--	R905	--	14	18
	12	-7.1	--	--	E902	S434, V437, V472, S474, D555, I585	--	--	--	--	6	19
Carbohydrazides	13	-7.1	R905	E476	N903, R905	V437, V472, S474, Q475, D555, I585	--	--	--	--	12	15
	14	-7.1	N903	--	R905	C431, S434, V437, V472, S474, D555, I585	--	--	--	--	7	20
	15	-7.6	--	--	R905	S434, S474, D555, I585	--	--	--	--	9	14
	16	-7.6	R905	--	E902	G430, S434, V437, V472, S474, D555, I585	--	--	--	--	5	19
	17	-7.4	--	S434	N903, T906	V437, V472, S474, D555, I585	--	--	--	--	9	14
	18	-7.3	--	S434	E902, N903, T906	V437, V472, S474, L553, L554, D555, I585	--	--	--	--	11	20
	19	-7.5	R905	V473, E476	--	G430, S434, V437, V472, S474, L554, D555, I585	--	--	--	--	7	22
	20	-7.7	--	--	E902, N903	V437, V472, S474, E476, D555, I585	--	--	--	--	11	21
	21	-7.4	N903	Y401	R905	Y401, S403, S434, V437, D555, I585	--	--	--	--	10	14
	22	-8.1	--	--	E902, N903	S434, V437, E476, D555, I585	--	--	R905	--	9	17
	23	-8.1	--	S434	E902, N903	S434, V437, D555, I585	--	--	R905	--	10	20
	24	-7.8	R905	S434, E476	R905, T906	Q475, S434, V437, S474, V437, I585	--	--	--	--	10	17
4'-O derivatives	26	-6.3	--	--	E902, T906	S434, V437, S474, E476, I585	--	--	--	--	12	15
	27	-6.3	--	--	E902, N903, R905, T906	S434, V437, S474, D555, I585	--	--	--	--	14	23
	28	-6.4	--	--	R905, T906	V437, S474, E476, D555, I585	--	--	--	--	9	16

Table S5. NBD2 residue interactions with top-ranked docked poses of all tested molecules and total number of contacts.

	Molecule Name	binding energy (kcal/mol)	Hydrogen Bonds		Hydrophobic Contacts		Pi-pi	T-stacking	Cation-pi	Salt Bridges	Contacts	
			ICH	NBD	ICH	NBD					ICH	NBD
REFS	BUM	-6.4	T263	--	A260, R262	E1119, I1121	--	--	R262	R262	20	5
	NPA	-7.2	A259, R262	--	A259, A260, R262	S1117, Q1118	--	--	--	R262	19	7
	Naringenin (**1**)	-6.5	T263	Q1118	R262, A260, I261, T811	--	--	--	R262	--	26	3
	7-OMe-Naringenin (**25**)	-6.2	T263	--	E256, A269	Q1118, E1119	--	--	--	--	17	6
Thioxantones	TX1	-6.2	T263	--	E256	Q1118, D1200	--	--	--	--	7	7
	TX2	-6.2	--	E1119	A260, R262	S1117, Q1118, E1119, A1205	--	--	--	--	9	8
	TX3	-6.2	R262	--	E256, A260, R262, V264	S1117, Q1118, P1120, I1121	--	--	--	--	22	12
	TX4	-6.2	--	I1121	E256, A259, A260, V264	S1117, Q1118, P1120	--	--	--	--	13	14
	TX5	-7.1	--	--	E256, A259, A260, R262, T263, V264	Q1118, E1119	--	--	--	E1119	12	7
	TX6	-6.5	T263	--	A260, I261, R262, T811, D805	--	--	--	R262	--	23	5
	TX7	-6.4	--	--	E256, A259, A260, I261, R262	Q1118, E1119	--	--	--	--	14	5
	TX8	-7.1	--	--	A260, I261, R262	Q1118, E1119, I1121, A1205, L1206	--	--	R262	D1200	15	9
Hydrazones	2	-6.8	--	--	I261, R262, D805	Y1044, T1046, C1074	--	--	R262	--	20	9
	3	-7.2	--	--	A259, A260, I261, R262, T263, T811	--	--	--	R262	--	30	3
	4	-6.5	T263	--	E255, E256, A259, A260	Q1118	--	--	--	--	14	12
	5	-6.3	T263	--	E255, E256, A259, A260	Q1118	--	--	--	--	14	9
	6	-7.0	--	--	A259, A260, I261, R262, T263, D805, T811	--	--	--	R262	--	33	1
	7	-6.7	L258, T811	--	I261, R262, A260, A259	--	--	--	R262	--	22	1
	8	-6.8	L258, T811	--	A259, A260, R262, T263, T811	T1046, D1200	--	--	R262	--	20	5
Azines	9	-6.9	T811	--	T811, D805, I261, A259, R262, T263	--	--	--	R262	--	29	2
	10	-7.0	--	--	A259, A260, I261, R262, T263, D805, T811	Q1118, D1200	--	--	--	--	32	5
	11	-6.9	T263	--	A260, R262, D805, T811	Y1044	--	--	R262	--	25	4
	12	-7.5	R262, T263	Q1118	A260, I261, R262, T811, T810	--	--	--	--	--	27	3
Carbohydrazides	13	-7.1	R262	--	A259, I261, R262, T263, D805, T811	--	--	--	R262	--	40	3
	14	-7.0	--	I1121	E256, A259, A260	S1117, Q1118, E1119, I1121	--	--	--	--	8	13
	15	-8.5	A259, T263, D805	--	A259, A260, I260, D805, T811	--	--	--	--	--	25	2
	16	-8.3	T263, D805	--	A260, I261, R262, D805, T811	G1073	--	--	R262	--	27	3
	17	-7.4	T263	E1119, D1200	A259, A260, I261, R262	Q1118	--	--	R262	--	21	11
	18	-7.1	T263	S1117, Q1118, D1200	A259, A260, E256, T263, V264	S1117, Q1118, P1120, I1121	--	--	--	--	15	19
	19	-7.7	R262, T263	E1119, D1200	A259, A260, R262	Q1118	--	--	R262	--	23	13
	20	-7.6	R262, T263	E1119, D1200	A259, A260, R262	Q1118, A1205	--	--	R262	--	22	14
	21	-7,0	--	E1119	E256, A259, A260, R262	Q1118, E1119	--	--	R262	--	15	7
	22	-7.4	F804	--	E255, A259, A260, D805, T811	--	--	--	--	--	19	3
	23	-7.5	--	--	A260, I261, T811	Q1118	--	--	--	--	17	6
	24	-8.4	T263	E1119, D1200	E256, A260, I261, R262	Q1118, E1119, I1121	--	--	R262	--	22	18
4'-O derivatives	26	-6.8	T263, F804	Q1118, D1200	A260, I261, R262, F804, D805, T810, T811	--	--	--	R262	--	40	4
	27	-6.6	T263	Q1118	A259, A260, I261, R262, F804, D805, T810, T811	--	--	--	R262	--	41	6
	28	-7.4	R262, T263, T811	--	A259, A260, I261, R262, T811	T1046, Q1118	--	--	--	--	41	9

Table S6: ΔRLU values for first-screening compounds.

Thioxanthone derivatives				
**Compound**	**ΔRLU**		**Compound**	**ΔRLU**
**BASAL**	3000		**TX4**	19000
**VERAPAMIL**	23667		**TX5**	19333
**TX1**	22667		**TX6**	21000
**TX2**	17667		**TX7**	19000
**TX3**	32333		**TX8**	16500

Flavanone derivatives				
**Compound**	**ΔRLU**		**Compound**	**ΔRLU**
**BASAL**	3000		**15**	12333
**VERAPAMIL**	15667		**16**	7000
**1**	12000		**17**	6333
**2**	13333		**18**	10000
**3**	8667		**19**	10000
**4**	10667		**20**	20000
**5**	7333		**21**	n.d. *
**6**	12333		**22**	8667
**7**	17000		**23**	6333
**8**	12667		**24**	6333
**9**	13000		**25**	9667
**10**	12667		**26**	9667
**11**	11667		**27**	3667
**12**	8667		**28**	1667
**13**	8000		**BUM**	7300
**14**	15000		**spiropedroxodiol**	11967

* not determined due to solubility issues.

Figure S1: Normalized RLU values for first-screening of compounds (all flavonoids, including BUM, verapamil and spiropedroxodiol).

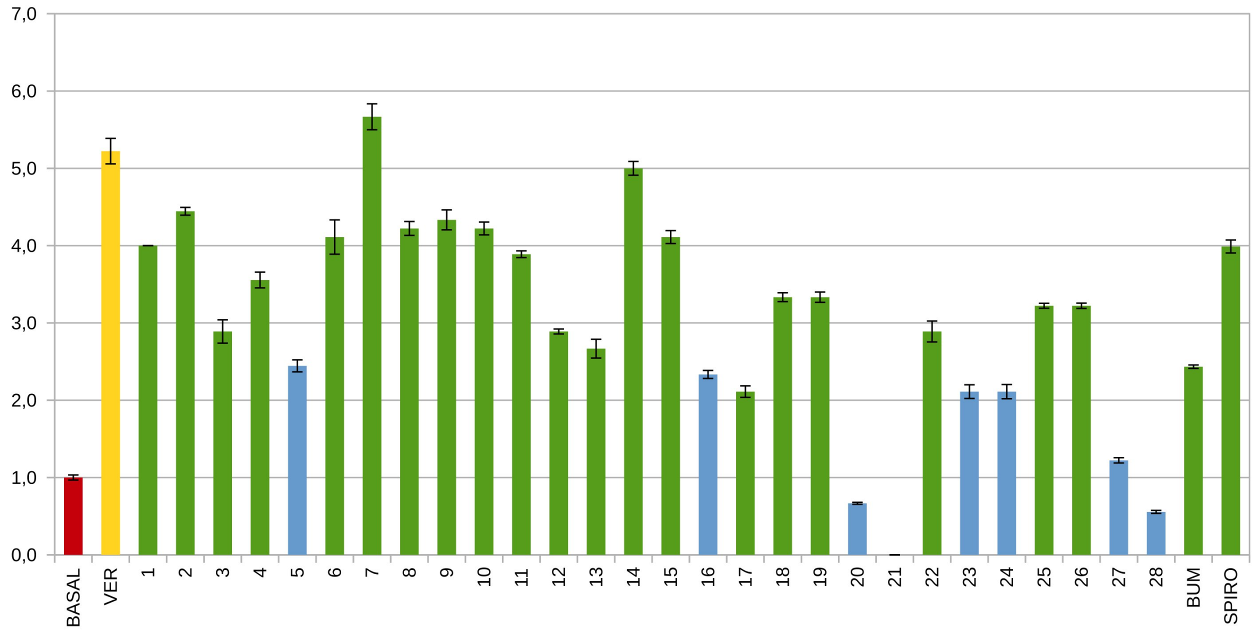

Figure S2: Normalized RLU values for first-screening of compounds (all compounds, thioxanthones).

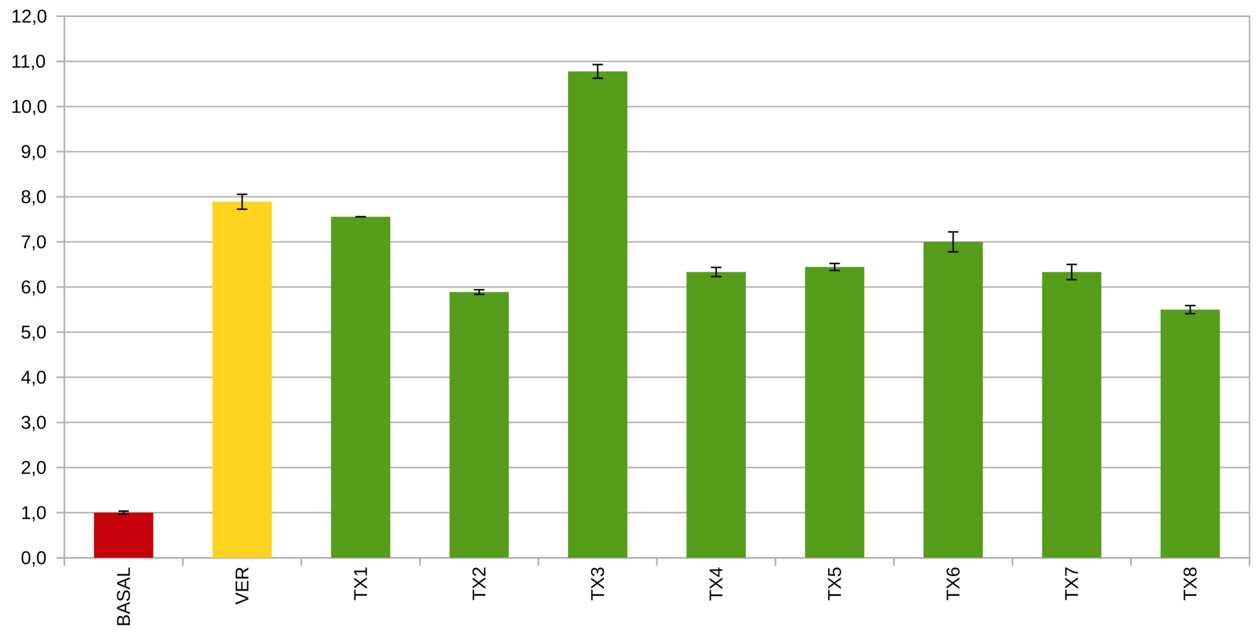

Table S7. Protein-residue contact frequencies, residue contributions to binding and hydrogen-bonding for the compound 23 after MD simulations.

Residue	*g_contacts*	
	n=3	***n*=2**
GLU-255	— *	0.343854
GLU-256	0.407123	—
ALA-259	0.437670	0.430233
ALA-260	0.213392	0.156146
ILE-261	0.136818	0.284053
ARG-262	0.418444	0.539867
PHE-804	—	0.501661
ASP-805	—	0.602990
THR-811	—	0.403655
THR-815	—	0.328904
TYR-1044	0.256382	0.453488
SER-1077	0.314192	—
SER-1117	0.515034	—
GLN-1118	0.768397	—
GLU-1119	0.947225	—
GLU-1201	0.324018	—

* contact frequency below 0.10

Residue	*g_mmpbsa*		
	n=3	***n*=2**	
LEU-258	-1.35	-7.81	kJ/mol
ALA-259	-9.31	-6.50	kJ/mol
ALA-260	-5.52	-1.45	kJ/mol
ILE-261	-2.08	-5.38	kJ/mol
PHE-804	-0.90	-7.05	kJ/mol
THR-811	-1.03	-6.12	kJ/mol
GLN-1118	-5.22	-0.13	kJ/mol

**Hydrogen-bond network**			
	n=3	***n*=2**	
lifetime	1274	1100	ps
ΔG	-21.894	-21.887	kJ/mol
<*N*>	1.503	0.284	per frame

REFERENCES

(1) Conseil, G.; Baubichon-Cortay, H.; Dayan, G.; Jault, J.-M.; Barron, D.; Di Pietro, A. Flavonoids: A Class of Modulators with Bifunctional Interactions at Vicinal ATP- and Steroid-Binding Sites on Mouse P-Glycoprotein. *Proc Natl Acad Sci USA* **1998**, *95(17)*, 9831–9836. https://doi.org/10.1073/pnas.95.17.9831.

(2) Ferreira, R. J.; Baptista, R.; Moreno, A.; Madeira, P. G.; Khonkarn, R.; Baubichon-Cortay, H.; dos Santos, D. J.; Falson, P.; Ferreira, M.-J. U. Optimizing the Flavanone Core toward New Selective Nitrogen-Containing Modulators of ABC Transporters. *Future Med Chem* **2018**, *10(7)*, 725–741. https://doi.org/10.4155/fmc-2017-0228.

(3) Kim, J. Y.; Henrichs, S.; Bailly, A.; Vincenzetti, V.; Sovero, V.; Mancuso, S.; Pollmann, S.; Kim, D.; Geisler, M.; Nam, H-G Identification of an ABCB/P-glycoprotein-specific inhibitor of auxin transport by chemical genomics. *J Biol Chem* **2010**, *285*, 23309–23317. https://doi.org/10.1074/jbc.M110.105981

(4) *Molecular Operating Environment* (MOE) v2019.01. Chemical Computing Group Inc, 1010 Sherbooke St. West, Suite #910, Montreal, QC, Canada, H3A 2R7

(5) Halgren, T. A. MMFF VI. MMFF94s option for energy minimization studies. *J Comput Chem* **1999**, *20*, 720–729. https://doi.org/10.1002/(SICI)1096-987X(199905)20:7<720::AID-JCC7>3.0.CO;2-X

(6) Schüttelkopf, A.W.; van Aalten, D. M. F. PRODRG: a tool for high-throughput crystallography of protein-ligand complexes. *Acta Crystallogr D Biol Crystallogr* **2004**, *60*, 1355–63. https://doi.org/10.1107/S0907444904011679

(7) Schmid, N.; Eichenberger, A. P.; Choutko, A.; Riniker, S.; Winger, M.; Mark, A. E.; van Gunsteren, W. F. Definition and testing of the GROMOS force-field versions 54A7 and 54B7. *Eur Biophys J* **2011**, *40*, 843–56. https://doi.org/10.1007/s00249-011-0700-9

(8) Lemkul, J.A.; Allen, W.J.; Bevan, D.R. Practical considerations for building GROMOS-compatible small-molecule topologies. *J Chem Inf Model* **2010**, *50*, 2221–2235. https://doi.org/10.1021/ci100335w

(9) Wang, J.; Wang, W.; Kollman, P. A.; Case, D. A. Automatic atom type and bond type perception in molecular mechanical calculations. *J Mol Graph Model* **2006**, *25*, 247–260. https://doi.org/10.1016/j.jmgm.2005.12.005

(10) Bonito, C. A.; Ferreira, R. J.; Ferreira, M.-J. U.; Gillet, J.-P.; Cordeiro, M. N. D. S.; dos Santos, D. J. V. A. Theoretical Insights on Helix Repacking as the Origin of P-Glycoprotein Promiscuity. *Sci Rep* **2020**, *10(1)*, 9823. https://doi.org/10.1038/ s41598-020-66587-5.

(11) Abraham, M.J.; Murtola, T.; Schulz, R.; Páll, S.; Smith, J. C.; Hess, B.; Lindahl, E. GROMACS: High performance molecular simulations through multi-level parallelism from laptops to supercomputers. *SoftwareX* **2015**, *1–2,* 19–25. https://doi.org/10.1016/j.softx.2015.06.001

(12) Kohlbacher, O.; Lenhof, H. P. BALL--rapid software prototyping in computational molecular biology. Biochemicals Algorithms Library. *Bioinformatics* **2020**, *16*, 815–824. https://doi.org/10.1093/bioinformatics/16.9.815

(13) Trott, O.; Olson, A. J. AutoDock Vina: improving the speed and accuracy of docking with a new scoring function, efficient optimization, and multithreading. *J Comput Chem* **2010**, *31*, 455–461. https://doi.org/10.1002/jcc.21334

(14) Ferreira, R. J.; Gajdács, M.; Kincses, A.; Spengler, G.; dos Santos, D. J. V. A.; Ferreira, M.-J. U. Nitrogen-Containing Naringenin Derivatives for Reversing Multidrug Resistance in Cancer. *Bioorg Med Chem* **2020**, *28(23)*, 115798. https://doi.org/ 10.1016/ j.bmc.2020.115798.

(15) Durães, F; Palmeira, A; Cruz, B; Freitas-Silva, J.; Szemerédi N.; Gales, L.; Costa, P. M.; Remião, F.; Silva, R.; Pinto, M.; Spengler, G.; Sousa, E. Antimicrobial Activity of a Library of Thioxanthones and Their Potential as Efflux Pump Inhibitors. *Pharmaceuticals* **2021**, *14(6)*, 572. https://doi.org/ 10.3390/ph14060572

(16) Morris, G. M.; Huey, R.; Lindstrom, W.; Sanner, M. F.; Belew, R. K.; Goodsell, D. S.; Olson, A. J. AutoDock4 and AutoDockTools4: Automated docking with selective receptor flexibility. *J Comput Chem* **2009**, *30*, 2785–2791. https://doi.org/10.1002/jcc.21256

(17) Durrant, J.D. & McCammon, J. A. BINANA: a novel algorithm for ligand-binding characterization. *J Mol Graph Model* **2001**, *29*, 888–893. https://doi.org/10.1016/j.jmgm.2011.01.004

(18) Wallace, A.C.; Laskowski, R. A.; Thornton, J. M. LIGPLOT: a program to generate schematic diagrams of protein-ligand interactions. *Protein Eng* **1995**, *8*, 127–134. https://doi.org/10.1093/protein/8.2.127

(19) Kumari, R.; Kumar, R.; Lynn, A. g_mmpbsa —A GROMACS Tool for High-Throughput MM-PBSA Calculations. *J Chem Inf Model* **2014**, *54*, 1951–1962. https://doi.org/10.1021/ci500020m

(20) Blau, C. & Grubmuller, H. g_contacts: Fast contact search in bio-molecular ensemble data.

Comput. Phys. Commun. **2013***, 184*, 2856–2859. https://doi.org/10.1016/j.cpc.2013.07.018

(21) Bussi, G.; Donadio, D.; Parrinello, M. Canonical sampling through velocity rescaling. *J Chem Phys* **2007**, *126,* 14101–14107. https://doi.org/10.1063/1.2408420

(22) Hoover, W. Canonical dynamics: Equilibrium phase-space distributions. *Phys Rev A* **1985**, *31*, 1695–1697. https://doi.org/10.1103/PhysRevA.31.1695

(23) Nosé, S.; Klein, M. L. Constant pressure molecular dynamics for molecular systems. *Mol Phys* **1983**, *50*, 1055–1076. https://doi.org/10.1080/00268978300102851

(24) Parrinello, M. Polymorphic transitions in single crystals: A new molecular dynamics method. *J Appl Phys* **1981**, *52*, 7182–7190. https://doi.org/10.1063/1.328693

(25) Hess, B.; Bekker, H.; Berendsen, H. J.; Fraaije, J. G. E. M. LINCS: A linear constraint solver for molecular simulations. *J Comput Chem* **1997**, *18*, 1463–1472. https://doi.org/10.1002/(SICI)1096-987X(199709)18:12<1463::AID-JCC4>3.0.CO;2-H

(26) Hess, B. P-LINCS: A Parallel Linear Constraint Solver for Molecular Simulation. *J Chem Theory Comput* **2008**, *4*, 116–122. https://doi.org/10.1021/ct700200b

(27) Miyamoto, S.; Kollman, P. A. Settle: An analytical version of the SHAKE and RATTLE algorithm for rigid water models. *J Comput Chem* **1992**, *13*, 952–962. https://doi.org/10.1002/jcc.540130805

(28) Darden, T.; York, D.; Pedersen, L. Particle mesh Ewald: An N·log(N) method for Ewald sums in large systems. *J Chem Phys* **1993**, *98*, 10089–10092. https://doi.org/10.1063/1.464397

(29) Essmann, U.; Perera, L.; Berkowitz, M. L. A smooth particle mesh Ewald method. *J Chem Phys* **1995**, *103*, 8577–8593. https://doi.org/10.1063/1.470117

(30) Pronk, S.; Páll, S.; Schulz, R.; Larsson, P.; Bjelkmar, P.; Apostolov, R.; Shirts, M. R.; Smith, J. C.; Kasson, P. M.; van der Spoel, D.; Hess, B.; Lindahl, E. GROMACS 4.5: a high-throughput and highly parallel open source molecular simulation toolkit. *Bioinformatics* **2013**, *29*, 845–854. https://doi.org/10.1093/bioinformatics/btt055

(31) Ferreira, R. J.; Kincses, A.; Gajdács, M.; Spengler, G.; dos Santos, D. J. V. A.; Molnár, J.; Ferreira, M.-J. U. Terpenoids from Euphorbia Pedroi as Multidrug-Resistance Reversers. *J Nat Prod* **2018**, *81(9)*, 2032–2040. https://doi.org/10.1021/acs. jnatprod.8b00326.

(32) Dongping, M.; Cali, J. J. Identify P-glycoprotein Substrates and Inhibitors with the Rapid HTS

Pgp-Glo™ Assay System. *Promega Notes* **2007**, *96*, 11–14

(33) Ambudkar, S. V.; Dey, S; Hrycyna, C. A.; Ramachandra, M.; Pastan, I.; Gottesman, M. M. Biochemical, cellular, and pharmacological aspects of the multidrug transporter. *Annu Rev Pharmacol Toxicol* **1999** *39*, 361–398. https://doi.org/10.1146/annurev.pharmtox.39.1.361

(34) Litman, T.; Skovsgaard, T.; Stein, W. D. Pumping of drugs by P-glycoprotein: a two-step process? *J Pharmacol Exp Ther* **2003**, *307*, 846–853. https://doi.org/10.1124/jpet.103.056960

(35) Borges-Walmsley, M.I.; McKeegan, K. S.; Walmsley, A. R. Structure and function of efflux pumps that confer resistance to drugs. *Biochem J* **2003**, *376*, 313–338. https://doi.org/10.1042/BJ20020957
